# Reduction of β-amyloid pathology by celastrol in a transgenic mouse model of Alzheimer's disease

**DOI:** 10.1186/1742-2094-7-17

**Published:** 2010-03-08

**Authors:** Daniel Paris, Nowell J Ganey, Vincent Laporte, Nikunj S Patel, David Beaulieu-Abdelahad, Corbin Bachmeier, Amelia March, Ghania Ait-Ghezala, Michael J Mullan

**Affiliations:** 1 The Roskamp Institute, 2040 Whitfield Avenue, Sarasota, FL 34243, USA

## Abstract

**Background:**

Aβ deposits represent a neuropathological hallmark of Alzheimer's disease (AD). Both soluble and insoluble Aβ species are considered to be responsible for initiating the pathological cascade that eventually leads to AD. Therefore, the identification of therapeutic approaches that can lower Aβ production or accumulation remains a priority. NFκB has been shown to regulate BACE-1 expression level, the rate limiting enzyme responsible for the production of Aβ. We therefore explored whether the known NFκB inhibitor celastrol could represent a suitable compound for decreasing Aβ production and accumulation *in vivo*.

**Methods:**

The effect of celastrol on amyloid precursor protein (APP) processing, Aβ production and NFκB activation was investigated by western blotting and ELISAs using a cell line overexpressing APP. The impact of celastrol on brain Aβ accumulation was tested in a transgenic mouse model of AD overexpressing the human APP695sw mutation and the presenilin-1 mutation M146L (Tg PS1/APPsw) by immunostaining and ELISAs. An acute treatment with celastrol was investigated by administering celastrol intraperitoneally at a dosage of 1 mg/Kg in 35 week-old Tg PS1/APPsw for 4 consecutive days. In addition, a chronic treatment (32 days) with celastrol was tested using a matrix-driven delivery pellet system implanted subcutaneously in 5 month-old Tg PS1/APPsw to ensure a continuous daily release of 2.5 mg/Kg of celastrol.

**Results:**

*In vitro*, celastrol dose dependently prevented NFκB activation and inhibited BACE-1 expression. Celastrol potently inhibited Aβ_1-40 _and Aβ_1-42 _production by reducing the β-cleavage of APP, leading to decreased levels of APP-CTFβ and APPsβ. *In vivo*, celastrol appeared to reduce the levels of both soluble and insoluble Aβ_1-38_, Aβ_1-40 _and Aβ_1-42_. In addition, a reduction in Aβ plaque burden and microglial activation was observed in the brains of Tg PS1/APPsw following a chronic administration of celastrol.

**Conclusions:**

Overall our data suggest that celastrol is a potent Aβ lowering compound that acts as an indirect BACE-1 inhibitor possibly by regulating BACE-1 expression level via an NFκB dependent mechanism. Additional work is required to determine whether chronic administration of celastrol can be safely achieved with cognitive benefits in a transgenic mouse model of AD.

## Background

Alzheimer's disease (AD) is an ever-increasing health concern among the aging population and is the most common form of dementia affecting more than 25 million individuals worldwide. While the cause of the disease is uncertain, there are two major neuropathological hallmarks present in the brains of AD patients: the extracellular senile plaques and the intracellular neurofibrillary tangles. Neurofibrillary tangles contain hyperphosphorylated microtubule-associated protein tau, while senile plaques contain a core of β-amyloid (Aβ) peptide. Current treatments for AD include cholinesterase inhibitors and glutamate antagonists. Although useful, these symptomatic treatments do not stop the disease process or prevent neuronal degeneration. There is an on-going need for the development of new treatments for AD. Although the central role of Aβ remains to be proven in clinical trials, data accumulated during the past two decades place Aβ peptides and in particular soluble forms of the peptide as being the main molecule initiating the pathological cascade that eventually leads to AD [1]. Consequently, significant resources have been allocated to identify and develop treatment strategies that alter the metabolism of Aβ. To this end, the discovery of new pharmaceutical entities that have Aβ-lowering ability remains a priority.

Aβ peptides are derived from the sequential proteolysis of the β-amyloid precursor protein (APP) by β- and γ-secretases. The major β-secretase is an aspartyl protease termed BACE-1 (β-site APP cleaving enzyme) [2-4]. BACE-1 cleaves APP within the extracellular domain of APP, resulting in the secretion of the large ectodomain (APPsβ) and generating a membrane-tethered C-terminal fragment CTFβ or C99 which serves as a substrate for γ-secretase [5]. The multimeric γ-secretase complex cleaves at multiple sites within the transmembranous CTFβ generating C-terminally heterogeneous Aβ peptides ranging between 38 to 43 amino-acid residues in length that are secreted [6]. In addition to BACE-1 and γ-secretase, APP can be cleaved by α-secretase within the Aβ domain between Lys^16 ^and Leu^17^, releasing APPsα and generating CTFα or C83 which is further cleaved by γ-secretase to generate an N-terminally truncated Aβ termed p3. Genetic ablation of BACE-1 completely abolishes Aβ production, establishing BACE-1 as the major neuronal enzyme responsible for initiating the amyloidogenic processing of APP [7].

Given that BACE-1 is the initiating enzyme in Aβ generation, it is considered a prime target for lowering Aβ levels in the treatment and/or prevention of AD. BACE-1 expression is tightly regulated at both the transcriptional and translational level [8]. The BACE-1 gene spans approximately 30 kilobases on human chromosome 11q23.2 and includes 9 exons. The BACE-1 gene promoter lacks the typical CAAT and TATA boxes but contains a variety of transcription factor binding sites, including those for Sp1, GATA-1, AP1, CREB, HSF-1, STAT1, and NFκB, among others. It is likely that these sites influence transcriptional activity of the BACE-1 promoter. Interestingly, both the expression and activity of BACE-1 appear to be elevated in the cerebrospinal fluid and brains of AD patients [9,10] suggesting that BACE-1 expression is altered in AD and that therapeutic approaches aimed at regulating BACE-1 expression may be successful.

Neurons are believed to be the major source of Aβ and BACE-1. However, evidence is mounting that glia, and astrocytes in particular may produce significant levels of BACE-1 and Aβ, especially during inflammation. Glia out-number neurons by a factor of 10, so even a slight increase in glial BACE-1 expression might contribute substantially to cerebral Aβ and exacerbate AD pathology. NFκB is increased in AD and particularly in astrocytes, and it has been suggested that Aβ itself can trigger BACE-1 expression in glial cells via NFκB activation [11-15]. BACE-1 expression has been shown to be particularly elevated both in neurons and astrocytes around Aβ deposits in AD brains [16,17,9]. Thus, pathophysiological conditions leading to the activation of astrocytes may increase BACE-1 expression resulting in increased Aβ production which could exacerbate AD pathogenesis. Small increases in BACE-1 expression have been shown to result in sharply increased Aβ production [18]. Altogether these data suggest that lowering BACE-1 expression levels may have therapeutical potential for AD. We have therefore explored *in vitro *the effects of various known NFκB inhibitors as a way to reduce BACE-1 expression and shown that NFκB inhibitors reduce Aβ production by targeting the β-cleavage of APP [19]. In the present study, we investigated acute and chronic effects of celastrol, a potent natural triterpene NFκB inhibitor extracted from the roots of the Chinese "Thunder of God vine" (*Tripterygenium wilfordii*) on Aβ production and Alzheimer-like pathology in a transgenic mouse model of AD. Root extracts of the "Thunder of God vine" have been used as an anti-inflammatory remedy for centuries in China. Celastrol was selected over other known NFκB inhibitors for its ability to cross the blood brain barrier and offer neuroprotection in animal models of Parkinson's disease and Huntington's disease [20]. Previous work has revealed that celastrol can prevent neurodegeneration and extend the life span of a transgenic mouse model of amyotrophic lateral sclerosis [21]. Celastrol displays potent anti-inflammatory activities *in vivo *and has been shown, for example, to be beneficial against allergy-induced asthma [22] as well as rheumatoid arthritis [23] and to inhibit tumor cell invasion through suppression of NFκB signaling [24].

## Methods

### Cell culture and western-blotting experiments

NFκB activation was quantified using a stable NFκB luciferase reporter cell line of HEK293 cells with chromosomal integration of a luciferase reporter construct regulated by 6 copies of the NFκB response element (Panomics, CA, USA). Cells were grown in DMEM containing 10% serum, 1% penicillin/streptomycin/fungizone and 100 μg/ml of hygromycin B. Confluent cells were treated with 20 pg/ml of TNFα (Sigma, MO, USA) to induce NFκB activation and with a dose range of celastrol for 3 hours. Celastrol was purchased by Gaia Chemical Corporation (CT, USA). Luciferase activity was detected with the Luc-Screen Extended-Glow from Applied Biosystem (CA, USA) and a Synergy HT Biotek chemoluminescent reader (VT, USA). The medium surrounding the cells was collected and used to assess cytotoxicity using a Lactate Dehydrogenase (LDH) assay (Roche Diagnostics, Germany) according to the manufacturer's protocol. Similar experiments were also conducted with 100 ng/ml of phorbol 12-myristate 13 acetate (PMA) purchased from Sigma (MO, USA) instead of TNFa to induce NFκB activation (data not shown). HEK293 cells were used to assess the effect of celastrol on the activation of canonical members of the NFkB signaling pathway following PMA stimulation and on BACE-1 expression levels. Briefly, HEK293 cells were treated 100 ng/ml of PMA or a combination of PMA with celastrol (5 mM) for 15 minutes. Celastrol effects on RAF-1, MEK1/2, p44/42 MAPK and IKBa phosphorylation were monitored by western-blots as detailed below.

7 W CHO cells overexpressing wild-type human APP [25] were grown in DMEM (ATCC, VA, USA) containing 10% fetal bovine serum (Invitrogen, CA, USA), 1% penicillin/streptomycin/fungizone (Cambrex, ME, USA) and 0.3% Geneticin (Invitrogen, CA, USA) and used to determine the impact of celastrol on APP processing and Aβ production. Confluent cells were treated for 24 hours with different doses of celastrol.

Cellular proteins were extracted with 150 μL of ice-cold M-PER Reagent (Pierce Biotechnology, Rockford, IL, USA) containing 1 mM phenylmethanesulfonyl fluoride, 1× of protease cocktail inhibitor (Roche, Inc., USA) and 1 mM sodium orthovanadate. Samples were sonicated, denatured by boiling in Laemmli buffer (Bio-Rad, Hercules, CA, USA) and resolved onto 4-20% gradient polyacrylamide gels (Bio-Rad, Hercules, CA, USA). After electrotransfering onto polyvinylidene difluoride membranes, western-blots were immunoprobed with an anti-APP C-terminal (751-770) antibody (EMD Biosciences Inc., San Diego, CA, USA), with an anti-actin antibody (Chemicon, Temecula, CA, USA) used as a reference antibody to quantify the amount of proteins electrotransferred, with a BACE-1 antibody (Invitrogen, CA, USA), with a phospho-IKBα (ser32) antibody, with a phospho-NFκB p65 (ser 536) antibody, with a phospho-Raf-1 (Ser338) antibody, with a phospho-MEK1/2 and a phospho-p44/42 MAPK antibody using dilutions recommended by the manufacturers. All phospho-specific antibodies were purchased from Cell Signaling Technology Inc (MA, USA). Additionally, sAPPα was detected by Western-blot in the culture medium surrounding 7 W CHO cells using the antibody 6E10 (Signet Laboratories Inc., MA, USA) which recognizes the amino-acids 1-17 of Aβ and sAPPβ was detected in the culture medium using an anti-human sAPPβ antibody (Immuno-Biological Laboratories Co. Ltd., Gunma, Japan). APP CTF/actin, sAPPβ/sAPPα signals, phospho-IKBα/actin, phospho-NFκB p65/actin, phpspho-RAF-1/actin, phospho-MEK/actin, phospho-p44/42 MAPK/actin, BACE-1/actin signal intensity ratios were quantified by chemiluminescence imaging with the ChemiDocTM XRS (Bio-Rad, Hercules, CA, USA). Aβ 1-40 and Aβ 1-42 were quantified in the culture media of 7 W CHO cells using commercially available ELISAs following the manufacturer's recommendations (Invitrogen, CA, USA).

Short hairpin RNAs (shRNAs) were used to stably knock-down the cdc37 gene in HEK293 cells overexpressing APPsw cells. Three different cdc37 shRNAs and a scrambled control shRNA (control cells) were used for transfection. Cdc37 shRNAs cloned into the pRs vector were purchased from Origene (Rockville, MD, USA). Approximately one million HEK/APPsw cells detached with TripLE (Invitrogen, IL, USA) were mixed together with 10 μg of shRNA vectors in Bio-Rad (Hercules, CA, USA) gene pulser cuvettes and electroporated using a square wave protocol (110 V, one 25 ms pulse length using a Bio-Rad gene pulser). Forty-eight hours after, the culture media were replaced by selective media containing 6 μg/ml of puromycin for stable selection of transfected cells. Single colonies resistant to puromycin were isolated. Western-blots were run with cell lysates from these clones to confirm silencing of the cdc37 gene using a 1:1000 dilution of an anti-cdc37 (V367) antibody (Cell Signaling Technology Inc, MA, USA). BACE-1 expression and APP-CTF levels were monitored in cdc37 knock-down HEK293 APPsw cells and HEK293 APPsw cells transfected with a scrambled control shRNA vector as described above.

In order to inhibit HSP90, HEK293 APPsw overexpressing cells were treated for 24 hours with 10 and 20 μM of gedunin (Tocris, MI, USA), a known HSP90 inhibitor which has been shown to inhibit HSP90 with an IC50 ranging from 3 μM to 8 μM in function of the cell type used [26]. Following 24 hours treatment with gedunin, cellular lysates were prepared and assayed for BACE-1 expression and APP-CTF levels by western blotting as described above.

The effect of celastrol on the disruption of the cdc37-HSP90 complex was tested in HEK293 cells overexpressing APPsw. Briefly, HEK293 APPsw cells were treated with PMA 100 ng/ml and celastrol 5 μM for 3 hours. Cellular lysates were prepared as described above and immunoprecipited overnight at 4°C with a 1:100 dilution of an HSP90 antibody (Cell Signaling Technology Inc, MA, USA). Protein A sepharose (GE Healthcare, NJ, USA) was used to pull down the immunoprecipitated complex which was denatured and analyzed by westernblotting for the presence of cdc37.

### Animals and treatments

All the experimentations involving mice were approved by the Institutional Animal Care and Use Committee of the Roskamp Institute. Transgenic mice overexpressing the human APP695sw mutation and the presenilin-1 mutation M146L (Tg PS1/APPsw) resulting in over production of human APP and Aβ [27] were used to assess the effect of celastrol on brain Aβ levels. These transgenic mice typically start to develop Aβ deposits by 4 month of age and display a significant amount of Aβ deposits by 6 months [27,28]. An acute treatment regimen was first tested by injecting intraperitoneally 35 week-old Tg PS1/APPsw (post plaque formation) with 1 mg/Kg body weight of celastrol (n = 7) or with 100 μL of the vehicle (50% DMSO in PBS, n = 7) daily for 4 days. One hour after the last injection, brains of the animals were collected, snap frozen in liquid nitrogen and stored at -80°C.

Custom made biodegradable pellets ensuring a continuous release of celastrol at 2.5 mg/Kg of body weight/day and placebo pellets were obtained from Innovative Research of America (FL, USA). Pellets were implanted subcutaneously in 5 month-old Tg PS1/APPsw mice (placebo n = 7; celastrol n = 7) when Aβ deposits are forming. After one month (32 days) of treatment, one brain hemisphere for each mouse was snap frozen in liquid nitrogen and stored at -80°C whereas the other hemisphere was fixed in 4% paraformaldehyde for 48 hours at 4°C prior to embedding in paraffin using a Tissue-Tek automated embedding system (Sakura, USA).

### Brain Aβ quantifications

Mice brains (not including the cerebellum) were homogenized by sonication in ice cold MPER reagent containing 1 mM PMSF, 100 mM sodium orthovanadate and 1× cocktail of protease inhibitors (Pierce protein research product, IL, USA). Brain homogenates were centrifuged at 4°C for 30 min at 15000 rpm. The supernatant containing soluble Aβ (GS) was collected and treated with 5 M guanidine isothiocyanate for 1 hour at room temperature. The pellet containing insoluble Aβ (GI) was dissolved and denatured in 5 M guanidine isothiocyanate. Protein concentrations were estimated in both fractions by the BCA method (Pierce, IL). For the mice that received an acute treatment with celastrol, Aβ 1-40 and Aβ 1-42 were quantified by ELISAs according to the manufacturer's recommendations (Invitrogen, CA, USA). For the mice that received the chronic celastrol treatment, Aβ 1-38, Aβ 1-40 and Aβ 1-42 were quantified by electrochemoluminescence in GI and GS fractions using multiplex Aβ assays according to the manufacturer's recommendations (Meso Scale Discovery, MD, USA). Aβ concentrations were calculated in pg/mg of protein and expressed as a % of Aβ obtained in the vehicle/placebo treated animals.

### Immunohistochemistry

Paraffin embedded brains were sagitally cut into 5 μm-thick sections with a microtome (2030 Biocut, Reichert/Leica, Germany). Sections were mounted on slides and air-dried. Prior to staining, sections were deparaffinized in xylene (2 × 5 min) and hydrated in graded ethanol (2 × 5 min in 100%, 5 min in 85%, 5 min 70%) to water. Endogeneous peroxidase activity was quenched with a 20-min-H2O2 treatment (0.3% in water) and after being rinsed, sections were incubated with blocking buffer (Protein Block Serum-free, DakoCytomation) for 20 min. The monoclonal antibody 4G8 was used to stain Aβ deposits at a 1:750-dilution and purchased from Signet Laboratories (MA, USA). CD45 was immunodetected using a 1:50-dilution of a rat monoclonal antibody (clone IBL-3/16) from Serotec (NC, USA) and used as a microglial marker as previously described [29]. The diluted antibodies were applied onto the sections overnight at 4°C and were detected using the Vectastain ABC (avidin-biotin-peroxidase complex) Elite kit (Vector Laboratories, CA). The labeling was revealed by incubating sections in 0.05 M Tris-HCl buffer (pH 7.6) containing 3,3'-diaminobenzidine (Sigma, MO) and H_2_O_2_. For each brain, 4 to 5 stained sections were used to perform the quantification of 4G8 burden and 3 sections were used for the quantification of CD45 burden. The stained area within particular regions (hippocampus, cortex) was quantified using Image-Pro Plus software (Media Cybernetics, MD). An average value was calculated for each brain area from individual mice and expressed as a percentage of the total brain area examined.

### Statistical analyses

For statistical analyses, ANOVA, post-hoc comparisons and *t*-tests were performed where appropriate using SPSS for Windows release 12.0.1.

## Results

### In vitro effects of celastrol

**Figure 1:**
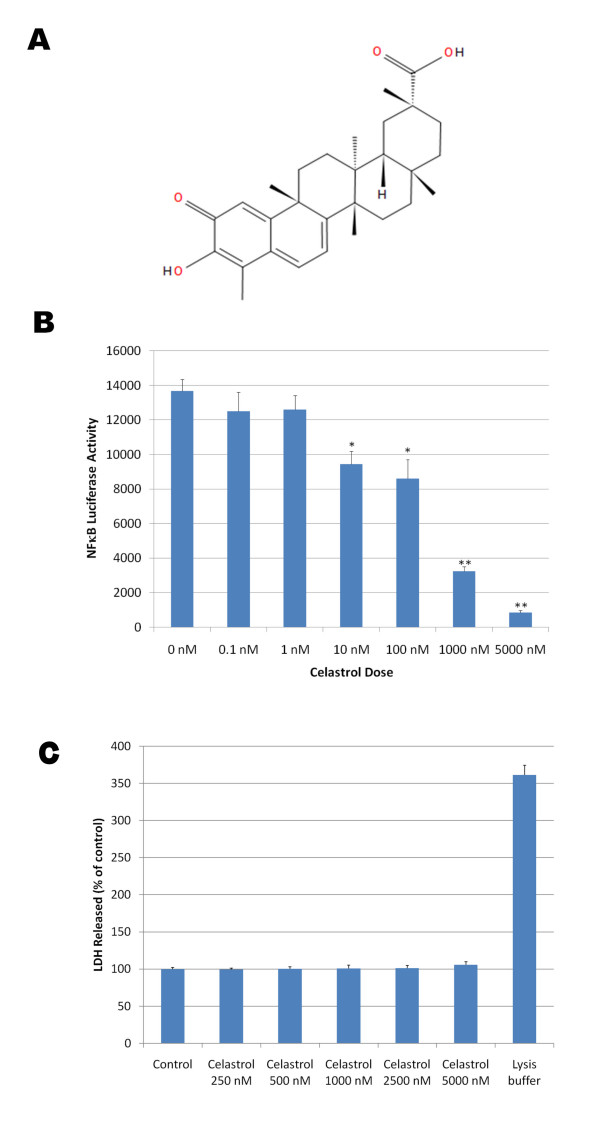
**A) Chemical structure of celastrol, an active ingredient of the "Thunder of God" medication which has been used as an anti-inflammatory remedy for centuries in China**. B) Dose dependent effect of celastrol on NFκB activation induced by TNFα in HEK293 cells stably expressing an NFκB luciferase reporter construct. Cells were co-treated with 20 pg/ml of TNFα and with a dose range of celastrol for 3 hours before measuring NFκB luciferase activity. ANOVA reveals a statistically significant main effect of celastrol dose on NFκB activation (P < 0.002). Post-hoc analysis show significant differences between NFκB activity in TNFα treated cells and cells co-treated with TNFα and celastrol for celastrol doses greater or equal to 10 nM (P < 0.005). C) The histogram represents the amounts of LDH released detected in the culture medium of HEK293 cells treated with a dose range of celastrol and with the MPER lysis buffer as a positive control (lysis buffer) to induce complete cellular lysis. ANOVA reveals no significant main effect of celastrol dose (P = 0.420) and a significant effect of the lysis buffer (P < 0.001) on LDH released.

**Figure 2:**
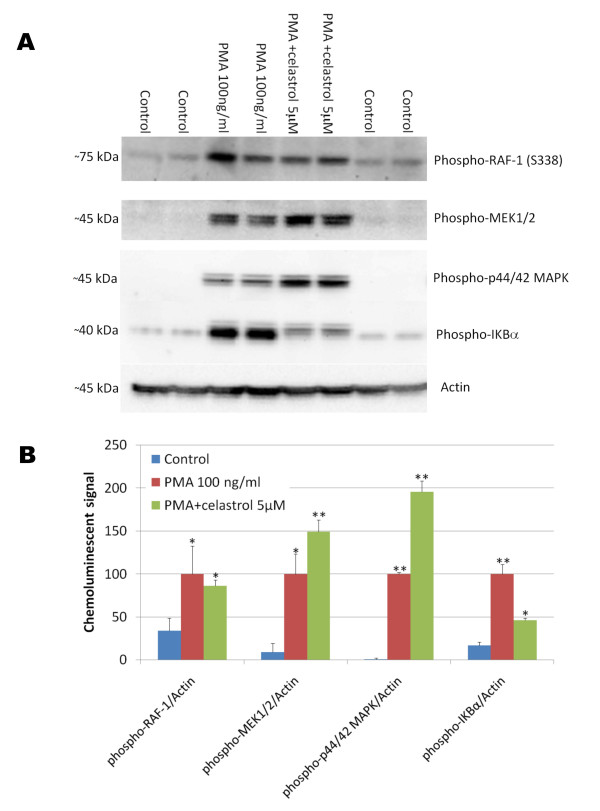
**Effect of celastrol on phorbol ester (PMA) induced phosphorylation of key members of the NFκB signaling pathway**. HEK293 cells overexpressing APPsw were treated for 15 minutes with PMA in the presence of 0 μM and 5 μM of celastrol. A) Western blots reveal that celastrol did not inhibit Raf-1 phosphorylation, MEK1/2 phosphorylation or p44/42 MAPK phosphorylation induced by PMA but prevented IKBα phosphorylation induced by PMA suggesting that celastrol is inhibiting IKK activity. B) Histogram representing the quantification of Raf-1 phosphorylation/actin, MEK1/2 phosphorylation/actin, phospho p44/42 MAPK/actin and IKBα phosphorylation/actin chemoluminescent signal. ANOVA shows a significant main effect of PMA on the phosphorylation of these different proteins (P < 0.05). Post-hoc comparisons reveal significant increase in phosphorylation after PMA treatment for Raf-1 (P < 0.04), MEK1/2 (P < 0.01), p44/42 MAPK (P < 0.001) and IKBα (P < 0.001) compared to the control conditions. Celastrol does not appear to prevent PMA induced Raf-1 phosphorylation (P = 0.619) but leads to a significant stimulation of MEK1/2 and p44/42 MAPK compared to PMA treatment alone (P < 0.02) and significantly inhibited IKBα phosphorylation compared to PMA treatment alone (P < 0.001).

Celastrol (3-Hydroxy-24-nor-2-oxo-1(10),3,5,7-friedelatetraen-29-oic acid) is a natural triterpene lactone epoxide compound (Figure 1A) extracted from the root bark of the Chinese medicine "Thunder of God Vine" which has been used for hundreds of years as a natural remedy for inflammatory conditions. Celastrol appears to potently inhibit NFκB activation induced by TNFα as monitored by measuring luciferase activity in a stable NFκB reporter HEK293 cell line with a half maximal inhibitory concentration (IC_50_) below 1 μM (Figure 1B) and no cytotoxicity was observed for doses of celastrol up to 5 μM (Figure 1C). Similar data were observed with celastrol when phorbol ester (PMA) was used in place of TNFα to induce NFκB signaling (data not shown). We investigated the possible effect of celastrol on some members of the canonical NFκB pathway following 15 minutes of stimulation with PMA. As expected, we observed that PMA rapidly stimulated the phosphorylation of RAF1, MEK1/2, p44/42 MAPK and IKBα (Figure 2). Interestingly, celastrol did not prevent PMA induced phosphorylation of RAF1, MEK1/2 and p44/42 MAPK but inhibited PMA induced IKBα phosphorylation suggesting that celastrol inhibits the activity of the IKK (IKappa B Kinase) signaling complex. The IKK signaling complex is also comprised of the catalytic components IKKα, IKKβ and of the regulatory subunit NEMO (NFκB essential modifying factor) which integrates upstream signals and leads to the phosphorylation of IKBα and NFκB. Subsequently, phosphorylated IKBα is ubiquitinated and rapidly degraded allowing the localization signal for NFκB nuclear translocation to be exposed.

**Figure 3:**
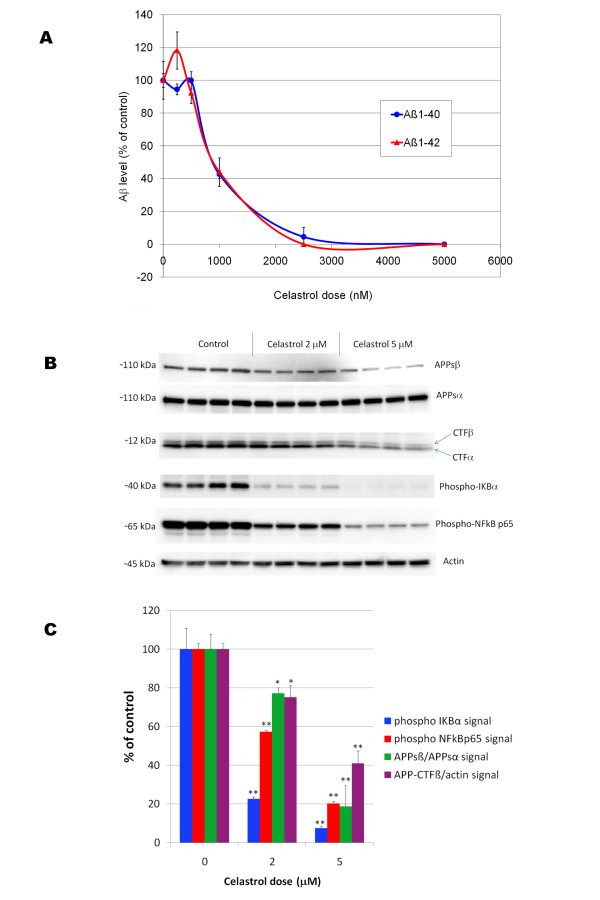
**A) Effect of celastrol on Aβ production in 7 W CHO cells overexpressing wild-type human APP**. Human Aβ was measured by ELISAs in the culture media surrounding the cells following 24 hours of treatment with different doses of celastrol. Dose response curves for both Aβ_1-40 _and Aβ_1-42 _were established revealing an IC_50 _of approximately 900 nM for celastrol. ANOVA reveals a significant main effect of celastrol on Aβ 1-40 (P < 0.001) and Aβ 1-42 production (P < 0.015). Post-hoc comparisons show statistically significant effects of celastrol at 1000 nM, 25000 nM and 5000 nM for both Aβ 1-40 and Aβ 1-42 (P < 0.001). B) Effect of celastrol on APP processing and NFκB in CHO cells overexpressing wild-type human APP. Celastrol dose dependently inhibited APPsβ secretion, APP-CTFβ level as well as NFκB p65 and IKBα phosphorylation. C) Histogram representing the quantification of APPsβ/APPsα, APP-CTFβ/actin as well as phospho-NFκB p65/actin and phospho-IKBα/actin chemoluminescent signals. ANOVA reveals a significant main effect of celastrol on APPsβ secretion, APP-CTFβ level, phospho-NFκB p65 and phospho-IKBα levels (P < 0.001). Post-hoc comparisons show a statistical significance for celastrol at 2 and 5 μM for all the parameters studied (P < 0.05) showing that celastrol dose dependently inhibits the β-cleavage of APP while suppressing NFκB activity. (* P < 0.05; ** P < 0.001).

**Figure 4:**
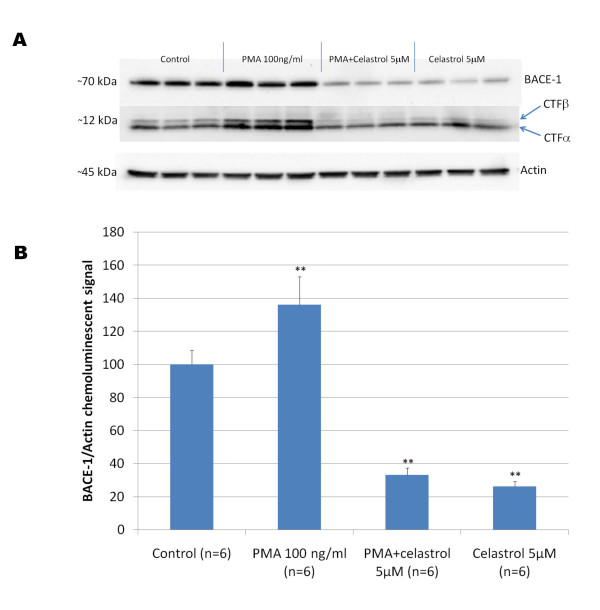
**A) Representative western-blot depicting the effect of celastrol on BACE-1 expression in HEK293 cells overexpressing APPsw**. Celastrol inhibited basal BACE-1 expression as well as the stimulation of BACE-1 expression induced by a 24 hours treatment with PMA. APP-CTFβ level is reduced when BACE-1 expression is inhibited by celastrol and APP-CTFβ level is increased when BACE-1 expression is stimulated by PMA. B) Histogram representing the quantification of BACE-1 expression in response to celastrol and PMA treatments. ANOVA reveals a significant main effect of PMA (P < 0.002) and of celastrol (P < 0.001) on BACE-1 expression. Post-hoc comparisons shows that PMA significantly stimulates BACE-1 level (P < 0.002) whereas celastrol significantly reduces BACE-1 expression (P < 0.001) compared to control condition and opposes BACE-1 stimulation induced by PMA (P < 0.001). (** P < 0.002).

We used 7 W CHO cells overexpressing wild-type human APP [25] to assess the effect of celastrol on Aβ production and APP processing. Following a 24 hours treatment, celastrol inhibited Aβ_1-42 _and Aβ_1-40 _production with an IC_50 _of approximately 900 nM (Figure 3A) in 7 W CHO cells overexpressing APP. Analysis of metabolites of the APP pathway reveals that celastrol impairs the cleavage of APP at the β-secretase cleavage site leading to a decreased sAPPβ and concomitant suppression of the APP-CTFβ (C99) fragment (Figure 3B). No significant effect of celastrol was observed on APPsα secretion indicating that celastrol does not affect the α-secretase cleavage of APP despite a reduction in APP-CTFα (C83) at 5 μM, therefore suggesting that celastrol may affect the stability or turn-over of C83 (Figure 3B). We did not find that celastrol directly affects BACE-1 activity in a cell free assay at doses inhibiting Aβ production in whole cells (data not shown). A dose dependent inhibition of IKBα and NFκB p65 phosphorylation was observed with celastrol confirming that celastrol inhibited basal NFκB activity in 7 W CHO cells overexpressing APP at doses affecting APP processing (Figure 3). We investigated the effect of celastrol and NFκB stimulation with PMA on BACE-1 expression in HEK293 cells overexpressing APPsw (Figure 4). Celastrol inhibited basal BACE-1 expression and opposed the stimulation of BACE-1 expression induced by PMA. In parallel with the reduction of BACE-1 expression induced by celastrol, a reduction in APP-CTFβ was observed. Moreover, an increased in APP-CTFβ and BACE-1 levels was observed when NFκB signaling was stimulated by PMA (Figure 4) suggesting that celastrol affects APP processing by regulating BACE-1 expression via an NFκB dependent mechanism. Similarly, using a neuronal like cell line (SHSY), we observed that celastrol can reduce BACE-1 expression and oppose PMA induced BACE-1 expression (data not shown).

**Figure 5:**
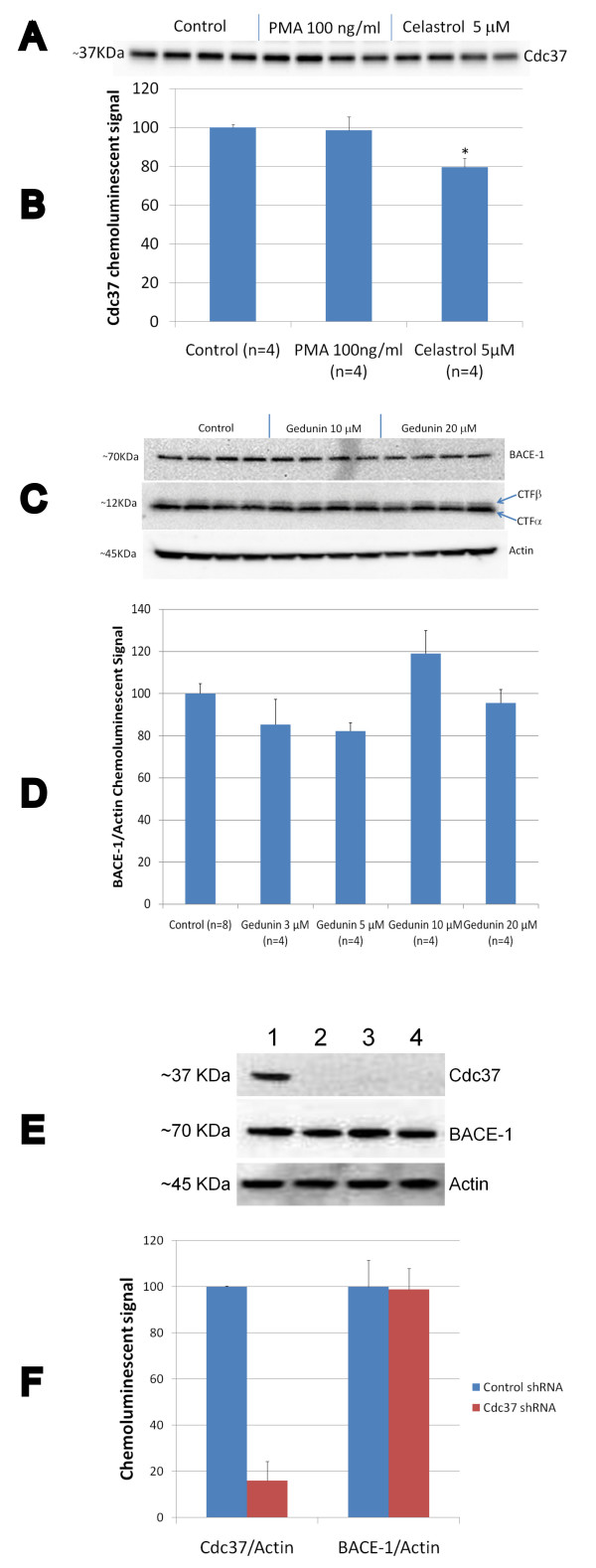
**Effect of celastrol and PMA on the formation of the HSP90-cdc37 complex in HEK293 APPsw cells**. A) Western-blot depicting the amount of cdc37 recovered in the HSP90 immunoprecipitate following treatment with celastrol and PMA. B) Histogram representing the quantification of cdc37 in the HSP90 immunoprecipitate. No significant effect of PMA was observed but a significant effect of celastrol was detected (P < 0.005) showing a slight reduction in the amount of cdc37 present in the HSP90 immunoprecipitates. C) Western-blots showing the effects of the HSP90 inhibitor gedunin on BACE-1 expression and APP processing in HEK293 APPsw cells. D) Histogram representing the quantification of BACE-1/Actin chemoluminescent signal showing that the gedunin treatment does not affect BACE-1 expression in HEK293 APPsw cells (ANOVA reveals no significant main effect of gedunin on BACE-1 level (P = 0.572)). E) Representative western-blot showing the level of cdc37 and BACE-1 expression in HEK293 APPsw cells that were knock-down for cdc37 using a shRNA approach for 3 different clones. 1) non silencing scrambled shRNA; 2) cdc37 shRNA clone 51G9; 3) cdc37 shRNA clone 97H1; 4) cdc37 shRNA clone 95C4. F) Histogram representing the quantification of cdc37 and BACE-1 expression for 9 different clones of HEK293 APPsw cells stably transfected with a silencing cdc37 shRNA vector (cdc37 shRNA) and 4 different clones of HEK293 APPsw stably transfected with a non silencing scrambled shRNA vector (control shRNA). Statistically significant inhibition of cdc37 expression (P < 0.001) and no effect on BACE-1 expression (P = 0.947) was observed in HEK293 APPsw cells knock-down for cdc37.

Since the formation of a transient complex between cdc37 and HSP90 may be required to allow IKK activation [30] and celastrol may interfere with HSP90 or cdc37 [31,32], we explored the effect of celastrol on the formation of this complex in HEK293 cells overexpressing APPsw. Data show that at a dose (5 μM) that fully inhibits NFκB activation and Aβ production, only a slight reduction of cdc37 was observed in the immunoprecipitated HSP90 complex (Figure 5A and 5B). No effect of the NFκB stimulator PMA was observed on the stability of this complex. We next inhibited HSP90 in HEK293 APPsw cells using gedunin, a known HSP90 inhibitor [26] which induces the degradation of HSP90-dependent client proteins similarly to other HSP90 inhibitors. Gedunin did not appear to affect BACE-1 expression or APP processing (Figure 5C and 5D). Moreover, we stably knock-down cdc37 expression in HEK293 APPsw cells using a shRNA approach and observed that cdc37 inhibition did not impact BACE-1 expression (Figure 5E and 5F) and APP processing (data not shown).

### Acute effect of celastrol on Aβ production in a transgenic mouse model of AD

**Figure 6:**
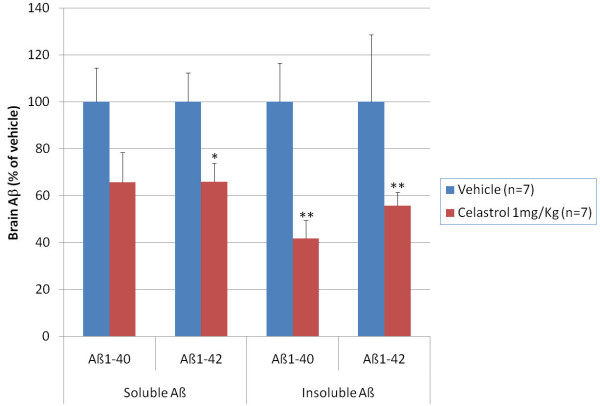
**Acute effects of celastrol on brain Aβ levels in 35 week-old Tg PS1/APPSw**. Mice were treated for 4 days with an intraperitoneal injection of celastrol (1 mg/Kg of body weight) or the vehicle only (placebo). Multivariate analysis reveals a significant main effect of celastrol on brain soluble and insoluble Aβ levels (P < 0.05). Statistically significant differences were observed for soluble Aβ 1-42 values (P < 0.03) between vehicle and celastrol treated mice and for insoluble Aβ 1-40 (P < 0.001) and Aβ 1-42 values (P < 0.001) between vehicle and celastrol treated mice. (* P < 0.05; ** P < 0.001).

We next investigated the effect of celastrol on brain Aβ levels using an acute dosage paradigm in 6-month old Tg PS1/APPsw mice. Tg PS1/APPsw mice were injected intraperitoneally, daily for 4 days, with 1 mg/Kg of body weight of celastrol or 100 μL of the vehicle only. One hour after the last injection, mice were humanely euthanatized and their brains collected. We assessed the β-amyloid lowering properties of celastrol by measuring the pools of soluble and insoluble Aβ species in celastrol and placebo treated mice. Following 4 days of treatment with 1 mg/Kg/day of celastrol, we observed a reduction in brain soluble Aβ 1-40 and Aβ 1-42 by approximately 40% whereas insoluble Aβ was reduced by approximately 50% compared to placebo treated animals (Figure 6).

### Chronic effect of celastrol on Alzheimer-like pathology in a transgenic mouse model of AD

**Figure 7:**
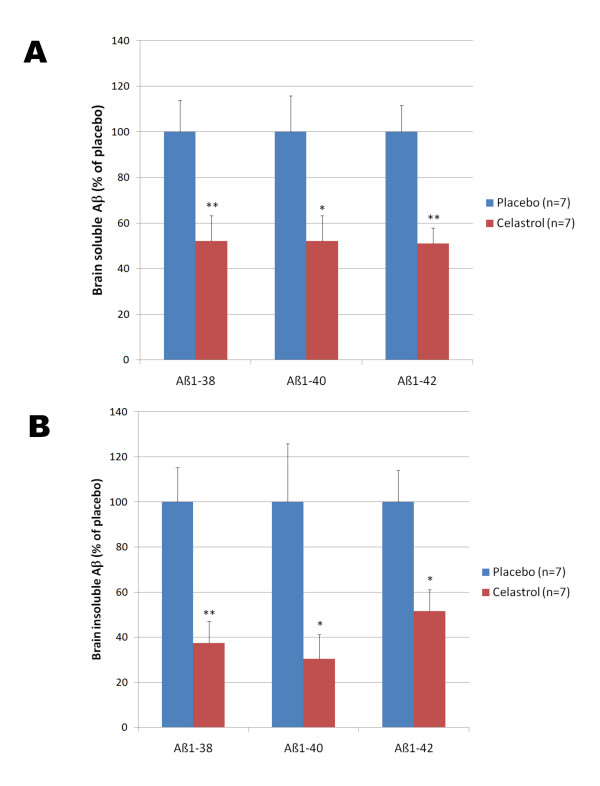
**Chronic effects of celastrol on brain Aβ levels**. Five month-old Tg PS1/APPsw mice were implanted subcutaneously with biodegradable placebo pellets and celastrol pellets ensuring a constant release of celastrol at a rate of 2.5 mg/Kg of body weight/day. After 32 days of treatment, brain Aβ levels were analyzed by electrochemoluminescence. A) Histogram representing the level of soluble Aβ 1-38, Aβ 1-40 and Aβ 1-42 quantified in the brain of placebo and celastrol treated Tg PS1/APPsw mice. B) Histogram representing the level of insoluble Aβ 1-38, Aβ 1-40 and Aβ 1-42 quantified in the brain of placebo and celastrol treated Tg PS1/APPsw mice. Multivariate analysis reveals a statistical significant differences between celastrol and placebo treated mice for the levels of brain soluble Aβ 1-38 (P < 0.005), Aβ 1-40 (P < 0.03), Aβ 1-42 (P < 0.007) and brain insoluble Aβ 1-38 (P < 0.004), Aβ 1-40 (P < 0.03), Aβ 1-42 (P < 0.02). (* P < 0.05); ** P < 0.01).

**Figure 8:**
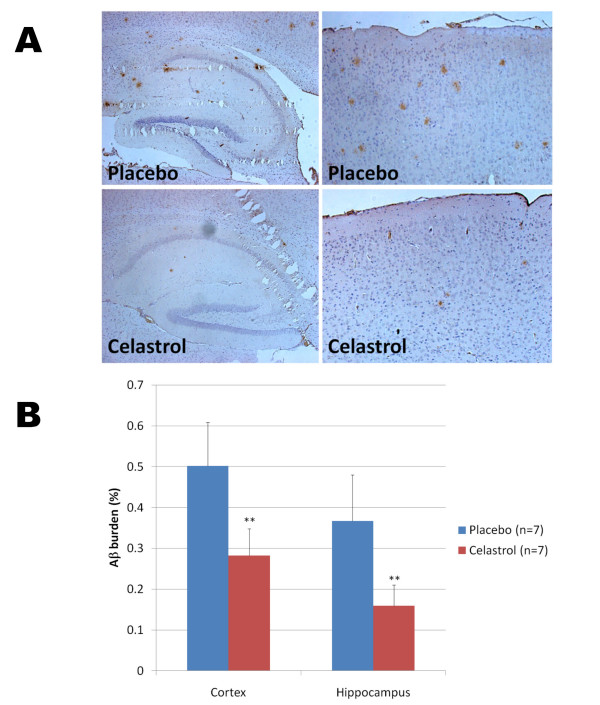
**Chronic effects of celastrol on Aβ plaque burden in Tg PS1/APPsw mice**. A) Representative photomicrographs (200× magnification) showing the extent of Aβ plaque burden detected by 4G8 immunostaining in the cortex (right panel) and hippocampus (left panel) of Tg PS1/APPsw mice (6 month-old) treated with biodegradable pellets of placebo or celastrol for a period of 32 days. B) Histogram representing the quantification of Aβ burden by image analysis in the cortex and hippocampus of Tg PS1/APPsw mice treated with placebo and celastrol pellets. Multivariate analysis reveals a statistically significant effect of celastrol treatment on Aβ plaque burden for the cortex (P < 0.002) and hippocampus (P < 0.004). (**P < 0.005).

**Figure 9:**
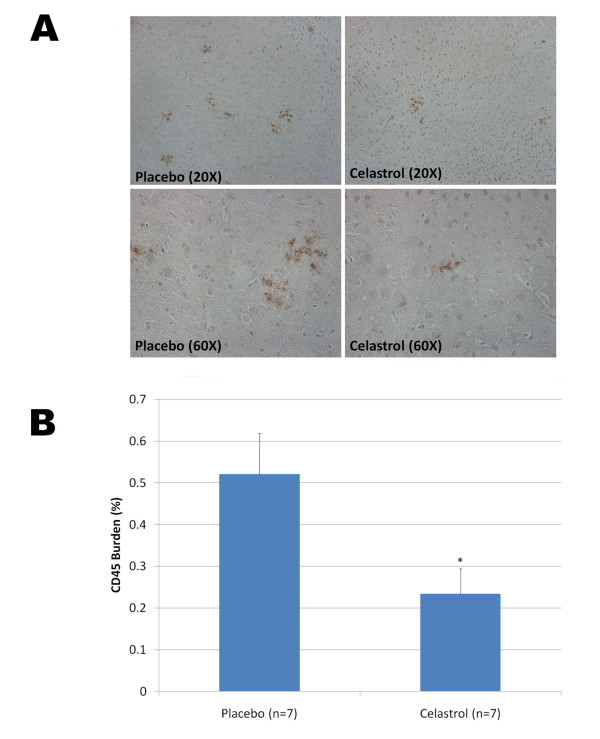
**Chronic effects of celastrol on microgliosis in Tg PS1/APPsw mice**. A) Representative photomicrographs (taken with a 20× and 60× objective providing a respective magnification of 200× and 600× respectively) depicting the presence of CD45 reactive microglia around Aβ deposits in Tg PS1/APPsw treated with a placebo and celastrol. B) Histogram representing the burden of activated microglia (CD45 positive) in the cortex of Tg PS1/APPsw mice treated with placebo and celastrol pellets. Statistically significant difference in microgliosis burden (P < 0.03) was observed between placebo and celastrol treated mice. (* P < 0.05).

To evaluate whether celastrol could affect Alzheimer-like pathology in Tg PS1/APPsw, we investigated the effects of a longer treatment with celastrol. We used a matrix-driven delivery pellet system implanted subcutaneously that was designed to ensure a continuous delivery of celastrol and avoid subjecting the animals to repeated injections and excessive handling. Tg PS1/APPsw mice were treated for one month with this pellet delivery system at a dosage of 2.5 mg of celastrol/Kg of body weight/Day. Analysis of brain Aβ levels by ELISAs revealed that celastrol reduced brain soluble Aβ 1-38, Aβ 1-40 and Aβ 1-42 by approximately 50% and the levels of insoluble Aβ 1-38, Aβ 1-40 and Aβ 1-42 by approximately 60% (Figure 7). To determine whether celastrol treatment affected β-amyloid plaque pathology, brain sections from Tg PS1/APPSw mice implanted with a placebo pellet and Tg PS1/APPSw mice implanted with a celastrol pellet were immunostained with the antibody 4G8 recognizing Aβ. A significant reduction in plaque burden (approximately 50%) in both the hippocampus and the cortex of celastrol treated animals compared to animals receiving the placebo was observed (Figure 8). We also estimated the impact of celastrol on microglial activation by measuring the percentage of CD45 immunostained area [29] on brain sections in placebo and celastrol treated animals. An approximate 50% reduction in microgliosis was observed in the cortex of celastrol treated animals compared to placebo treated animals (Figure 9).

## Discussion

NFκB has been shown to regulate BACE-1 expression level [11,17,13-15], the rate limiting enzyme responsible for the production of Aβ. For this reason, we investigated whether NFκB inhibition could lead to an inhibition of Aβ accumulation in a transgenic mouse model of AD. In this study, we selected the natural product celastrol over other known NFκB inhibitors because it is a potent NFκB inhibitor that has been shown to exert anti-inflammatory effects *in vivo *[33,34] and neuroprotective effects in animal models of Parkinson's disease and Hungtington's disease [20]. We first characterized the *in vitro *activity of celastrol towards Aβ production using a CHO cell line overexpressing Aβ. Celastrol dose dependently inhibited Aβ_1-40 _and Aβ_1-42 _with an IC50 comparable to its IC50 for inhibiting NFκB activation in response to TNFα (around 900 nM). Celastrol dose dependently reduced the production of APP-CTFβ and APPsβ confirming that celastrol impacted the β-cleavage of APP. Moreover, celastrol appeared to reduce BACE-1 expression and to oppose BACE-1 upregulation induced by NFκB stimulation.

We then investigated the impact of celastrol on Aβ production *in vivo *using a transgenic mouse model of AD. Following an acute treatment with celastrol in Tg PS1/APPsw, we observed a reduction in brain soluble and insoluble Aβ species. We used a biodegradable pellet delivery system implanted subcutaneously to assess the Aβ lowering effects of a chronic treatment with celastrol. After 1 month of treatment, pathological evaluation of the brains of celastrol treated Tg PS1/APPsw mice showed a 50% reduction in Aβ plaque burden in the hippocampus and cortex compared to placebo treated animals. Additionally, evaluation of activated microglia following CD45 immunostaining revealed that microgliosis was significantly reduced in celastrol treated animals compared to placebo treated mice paralleling the reduction of Aβ burden observed. We further characterized the β-amyloid lowering properties of celastrol by measuring the pools of soluble and insoluble Aβ species in celastrol and placebo treated mice. Data showed that this chronic treatment with celastrol in Tg PS1/APPsw mice reduced brain levels of soluble Aβ 1-38, Aβ 1-40 and Aβ 1-42 by approximately 50% and the levels of insoluble Aβ 1-38, Aβ 1-40 and Aβ 1-42 by approximately 60%.

The exact molecular mechanism of action of celastrol remains unclear. Celastrol has been suggested to be an ERK inhibitor and to inhibit of p44/42 MAPK phosphorylation [35], however our data show that celastrol inhibits NFκB activation without preventing the phosphorylation of MEK1/2 and its downstream target p44/42 MAPK when NFκB is stimulated with PMA, suggesting that celastrol is acting downstream of MAPK. Indeed, celastrol appears to oppose both IKBα and NFκB p65 phosphorylation which suggest that celastrol is inhibiting the IKK complex in agreement with previous studies showing that celastrol inhibits IKK activity [36]. It has been recently suggested that celastrol interacts with HSP90 and disrupts the formation of the cdc37-HSP90 complex [31]. However, NMR analyses of celastrol binding to cdc37 and HSP90 have suggested that celastrol directly interacts with ccd37 and not HSP90 [32]. Interestingly, the formation of a transient complex between cdc37 and HSP90 may be required to allow IKK activation [30] suggesting that celastrol may inhibit NFκB activation by preventing the formation of this complex. However, the disruption of the cdc37-HSP90 complex by celastrol requires 10 μM of celastrol [31] and at 5 μM (dose that fully inhibits Aβ production and NFκB activity *in vitro*), we only observed a modest reduction (20%) in the amount of cdc37 co-immunoprecipited with HSP90 suggesting that the effect of celastrol on Aβ production is unlikely to be mediated via a disruption of this complex. This is further substantiated by our observation that pharmacological inhibition of HSP90 does not significantly affect APP processing and BACE-1 expression, ruling out the possibility that the Aβ lowering properties of celastrol are mediated via HSP90 dependent events. Moreover, inhibition of cdc37 expression does not appear to affect APP processing and BACE-1 expression excluding cdc37 as a molecular target responsible for the Aβ lowering properties of celastrol. Celastrol has also been shown to inhibit lipidoperoxydation *in vitro *with an IC50 of 7 μM [37], which is 7-fold higher than it's IC_50 _for inhibiting Aβ production; it is therefore unlikely that the antioxidant properties of celastrol play a major role in the Aβ lowering activity of this compound. Overall, our *in vitro *data suggest that celastrol inhibits Aβ production by regulating BACE-1 expression level most likely via an NFκB dependent mechanism.

At doses similar to the doses of celastrol that we tested *in vivo*, celastrol has been shown to significantly inhibit NFκB activity in the brain, to reduce TNFα production and the expression of the CD40 ligand in astrocytes [20]. It remains possible that the *in vivo *Aβ lowering properties of celastrol may be the result of multiple mechanisms in addition to those affecting APP processing and BACE-1 expression as observed *in vitro*. For instance, celastrol may also lower brain Aβ levels by opposing inflammation, cytokines production and CD40 ligand expression, all of which are known to modulate the accumulation of Aβ in the brain [38-40]. So far, we have tested the effects of celastrol on brain Aβ accumulation when Aβ deposits are initially forming in a transgenic mouse model of AD, which could be regarded as an early intervention. It remains to be determined whether celastrol can display therapeutic efficacy when Aβ deposits are already highly abundant. Interestingly, celastrol has been shown to display neuroprotective effects in different animal models [20,21] and to improve memory, learning and psychomotor activity in rats [34]. Chronic effects of celastrol on memory impairment and tau pathology remain to be investigated in transgenic mouse models of AD to determine whether this compound may offer therapeutic benefits suitable for the treatment of AD.

## Conclusions

Our data suggest that celastrol is a potent Aβ lowering compound that may act in part by decreasing BACE-1 expression levels and Aβ production via an NFκB dependant mechanism. More experimentation is required to determine the exact mechanism of action responsible for the Aβ lowering properties of celastrol. It remains to be determined whether this compound can also improve cognition and tau pathology in transgenic mouse models of AD and whether this molecule displays the pharmacokinetic characteristics and toxicity profile required for a drug candidate.

## Competing interests

The authors declare that they have no competing interests.

## Authors' contributions

DP conceived of the study, developed the methodology for studying APP processing and NFκB activity, carried out the *in vivo *treatments with celastrol, participated in animal care, participated in data analysis and drafted the manuscript. NG and VL carried out the immunostaining studies. NP, DB, AM, CB participated in animal care, samples preparation and carried out some of the western blotting and ELISA studies. GA generated the cdc37 knock-down HEK293 APPsw cells and helped with cell culture experiments. MM helped write the manuscript and gave a critical analysis of the manuscript. All authors read and approved the final manuscript.
